# Brain-Derived Neurotrophic Factor Mitigates the Association Between Platelet Dysfunction and Cognitive Impairment

**DOI:** 10.3389/fcvm.2021.739045

**Published:** 2021-09-07

**Authors:** Jean-Christophe Bélanger, Véronique Bouchard, Jessica Le Blanc, Louisia Starnino, Mélanie Welman, Malorie Chabot-Blanchet, David Busseuil, Howard Chertkow, Bianca D'Antono, Marie Lordkipanidzé

**Affiliations:** ^1^Research Center, Montreal Heart Institute, Montreal, QC, Canada; ^2^Faculty of Pharmacy, Université de Montréal, Montreal, QC, Canada; ^3^Psychology Department, Faculty of Human Sciences, Université du Québec à Montréal, Montreal, QC, Canada; ^4^Montreal Health Innovations Coordinating Center, Montreal, QC, Canada; ^5^Baycrest Health Sciences, Rotman Research Institute, University of Toronto, Toronto, ON, Canada; ^6^Department of Medicine (Neurology), University of Toronto, Toronto, ON, Canada; ^7^Psychology Department, Faculty of Arts and Sciences, Université de Montréal, Montreal, QC, Canada

**Keywords:** platelets, BDNF, coronary artery disease, cognitive impairment, mediation model

## Abstract

**Background:** Platelet hyperactivity is deleterious in coronary artery disease (CAD), requiring lifelong antiplatelet therapy, and is associated with worse cognitive outcomes. Upon activation, platelets release Brain-Derived Neurotrophic Factor (BDNF), a neurotrophin protective against cognitive decline. Given these apparently opposing effects of platelet activation on cognitive health, we investigated whether BDNF levels intercede in the relationship between platelet activation and cognitive function; and whether this relationship is moderated by the presence of CAD.

**Methods:** In this cross-sectional study, 1,280 participants with (*n* = 673) and without CAD (*n* = 607) completed the Montreal Cognitive Assessment (MoCA). Plasma BDNF and soluble P-selectin (a marker of platelet activity) levels were assessed using multiplex flow cytometry.

**Results:** In a mediation model, platelet activity was correlated with higher plasma BDNF concentrations (b = 0.53, *p* < 0.0001). The relationship between sP-selectin and BDNF concentrations was stronger for individuals without CAD (b = 0.71, *p* < 0.0001) than for CAD participants (b = 0.43, *p* < 0.0001; *p*_interaction_ <0.0001). Higher BDNF concentrations were associated with higher MoCA scores (b = 0.26, *p* = 0.03). The overall effect of platelet activity on cognitive performance was non-significant (total effect: b = −0.12, *p* = 0.13), and became significant when accounting for BDNF as a mediating factor (direct effect: b = −0.26, *p* = 0.01). This resulted in a positive indirect effect of platelet activity (via BDNF) on MoCA scores (b = 0.14, CI 95% 0.02–0.30), that was smaller in CAD participants than in non-CAD participants [Δ −0.07 (95% CI −0.14 to −0.01)].

**Conclusions:** BDNF released from activated platelets could be a mitigating factor in a negative association between platelet activity and cognitive function.

## Introduction

While cardiovascular diseases and dementia share common risk factors, the mechanistic pathways underlying the heart-brain connection are not clearly understood (1). Platelet hyperactivity, one of the cardinal pathophysiological elements in coronary artery disease (CAD), is also an independent predictor of the severity of cognitive impairment (2–4). Indeed, platelet activation levels are negatively associated with cognitive health, and patients suffering from dementia often display higher platelet activation markers (2, 5, 6). Microvascular thrombosis could explain this association (7). However, as platelets contain an abundance of growth factors and vascular mediators released upon platelet activation at sites of vascular injury, platelet activity is central to vascular wound healing (8). Thus, platelets perform a delicate balancing act between thrombosis and vascular healing in the context of microvascular injury.

Interestingly, platelets are believed to be the largest peripheral reservoir of the Brain-Derived Neurotrophic Factor (BDNF), as evidenced by over 100-fold differences between plasma and serum BDNF levels (9), and it has been shown that platelet activation can release large quantities of BDNF into the bloodstream (10, 11). BDNF is a neurotrophin known to drive neuronal survival and differentiation, synaptic plasticity and connectivity in the brain, as well as memory formation and processing, learning, and cognition (12). In the largest study to prospectively associate circulating BDNF levels to cognitive health, Weinstein et al. have shown higher serum BDNF levels to be negatively associated with future occurrence of dementia and Alzheimer's disease (13).

The current study examined the interplay between platelet activity, BDNF, CAD, and cognitive health. We hypothesized that BDNF released by platelet activation would be a positive mediating factor in an otherwise negative relationship between platelet activation and cognitive function.

## Materials and Methods

### BEL-AGE Cohort

#### Participants Selection

This study is part of an ongoing prospective investigation (BEL-AGE) that seeks to examine the role of psychological burden on pathological aging.

Between September 2012 and June 2017, 1,280 men and women were recruited from the André and France Desmarais Hospital Cohort of the Montreal Heart institute (MHI), as previously described (14). Briefly, individuals were excluded from participating in BEL-AGE if they: (a) were diagnosed with bipolar disorder, schizophrenia, Alzheimer's disease, or irreversible dementia; (b) were diagnosed with a life-threatening degenerative disease, other than CAD, such as cancer (except skin cancer), AIDS, Creutzfeldt-Jakob disease, and amyotrophic lateral sclerosis; (c) were pregnant or breastfeeding; or (d) if a family member (including spouses) previously participated in BEL-AGE or was scheduled to participate. CAD at the time of enrollment into BEL-AGE was documented by the presence of coronary angiography (at least 50% stenosis), prior myocardial infarction, coronary artery bypass graft, or percutaneous coronary intervention. Absence of CAD was defined as no current or past history of CAD, angina, arrhythmia, congenital heart disease, heart failure, cardiomyopathy, and stroke. Medical history was self-reported and corroborated by consultation of participants' medical file in the case of CAD. The study was approved by the Research Ethics Committee of the Montreal Heart Institute [2011-202 (11–1313)] and all participants gave written informed consent.

#### Baseline Characteristics

At recruitment, data on sex, age, ethnicity, weight and height, years of education, smoking habits, hours spent exercising per week, personal and family medical history, as well as a current list of medication taken by the participant was collected.

#### Cognitive Testing

All participants were administered the Montreal Cognitive Assessment (MoCA) by a trained research assistant. The MoCA is a pragmatic, rapid and validated tool, that is sensitive to cognitive domains affected in early phases of vascular disease (15), and is recommended to evaluate overall cognitive functioning (16). Scored out of a possible total of 30 points, severe, moderate, mild, and no cognitive impairment, respectively reflect MoCA scores <10, between 10 and 17, between 18 and 26, and ≥26.The test was developed to screen milder forms of cognitive impairment through the assessment of six cognitive domains: executive functions; visuospatial abilities; memory; language; attention, concentration; and temporal and spatial orientation. To correct for education effects, one point was added for participants with ≤ 12 years of education (17). The MoCA score was used as a continuous variable in all analyses.

#### Blood Draw and Plasma Collection

Participants were scheduled for a laboratory appointment between 8:00 and 10:00 a.m. on a weekday to control for circadian rhythms. They were asked to abstain from eating, drinking (with the exception of water), smoking, and strenuous exercise for 12 h prior to testing. They were also asked to refrain from using illicit drugs or alcohol 24 h preceding their appointment but could continue taking medications as prescribed. Blood was drawn into EDTA-containing collection tubes (BD Vacutainer) and processed within 30 min of collection. The whole blood samples were centrifuged at 4°C at 1,500*g* for 15 min to obtain plasma. Plasma samples were aliquoted at 500 μl per tube and kept frozen at −80°C until analysis. Aliquots were used only once, i.e., a single freeze-thaw cycle, to avoid protein degradation. Thawed plasma samples were centrifuged at 4°C at 3,000*g* for 5 min to remove debris prior to quantification.

#### Assessment of Platelet Activation and Circulating BDNF Levels

Endogenous platelet activation was measured by assessing circulating levels of soluble P-selectin (sP-selectin) in plasma, as they are directly associated with platelet activation and correlate with major adverse cardiovascular events (18). The quantification of plasma sP-selectin and BDNF was carried out by flow cytometry (MACSQuant Analyzer 10 flow cytometer, Miltenyi Biotec, Germany) with multiplexed bead kits from AimPlex Biosciences (Pomona, CA, USA) as per the manufactures instructions. All samples were quantified in duplicate; if the coefficient of variation between duplicates exceeded 20%, samples were re-analyzed. Mean fluorescence intensity signals were acquired using the MACSQuantify software (Miltenyi Biotec, Germany) and analyzed using the FCAP array software (BD biosciences, San Jose, CA). Laboratory personnel were blinded to the clinical characteristics of the participants.

### Statistical Analysis

All data are expressed as the mean (standard deviation) for approximatively normally distributed data, and median (25th, 75th percentile) otherwise for continuous variables; and as frequency and percentage for categorical variables. BDNF and sP-selectin level were log-transformed prior analyses to satisfy the normality assumption. Continuous variables were compared between CAD status using the Student *T*-test or the Mann-Whitney *U*-test, and categorical variables were compared using the χ^2^ test. The association between sP-selectin and BDNF levels, sP-selectin and MoCA score, BDNF levels, and MoCA score was assessed using linear regression analyses, using sex and age as covariates.

In order to verify the moderating role of the CAD status, the potential interaction effects were tested using hierarchical regressions. To test for the mediating role of BDNF levels, we used the SPSS macro (v3.3) created by Preacher and Hayes (19). When significant mediation was established, conditional indirect effect procedures were used to determine whether mediation depended on CAD status (moderator). The Bootstrapping method was also used to obtain 95% confidence intervals. A two-sided *p*-value <0.05 was considered significant. All analyses were performed using SPSS 25.0 for MAC (SPSS Institute, Chicago, IL, USA).

## Results

### BEL-AGE Participant Characteristics

Of the 1,280 participants enrolled in this study, 673 presented with CAD and 607 were in the non-CAD group. Individuals with CAD were more often male and older (Table 1). As expected, cardiovascular risk factors, including smoking, dyslipidemia, hypertension, sedentary lifestyle, and diabetes were more prevalent in the CAD group. Most participants in the CAD group were taking antiplatelet, antihypertensive and cholesterol-lowering medications, and their estimated 10-year cardiovascular risk was higher than for non-CAD participants.

**Table 1:** Baseline characteristics.

**Variables**	**CAD (*n* = 673)**	**Non-CAD (*n* = 607)**	***P-*vaclue**	**Total (*n* = 1,280)**
**Demographics**
Age, years	66.3 (6.5)	64.3 (7.8)	<0.0001	65.3 (7.2)
Women, *n* (%)	158 (24)	352 (58)	<0.0001	510 (40)
Years of education	13.8 (3.6)	14.9 (3.5)	<0.0001	14.3 (3.6)
Physical exercise, hours per week	2 (0–5)	3 (1–5)	0.003	2.3 (0–5)
**Clinical characteristics**
BMI, kg/m^2^	30.1 (6.1)	29.1 (6.3)	0.007	29.6 (6.2)
Tobacco use, *n* (%)	94 (14)	36 (6)	<0.0001	130 (10)
Diabetes, *n* (%)	137 (20)	43 (7)	<0.0001	180 (14)
Dyslipidemia, *n* (%)	628 (93)	278 (46)	<0.0001	906 (71)
Hypertension, *n* (%)	465 (69)	203 (33)	<0.0001	668 (52)
10-year CV risk, %	24.4 (14.5)	17.6 (11.7)	<0.0001	21.1 (13.7)
MoCA score	26 (24–28)	27 (25–29)	<0.0001	27 (25–28)
No impairment, *n* (%)	322 (48)	376 (62)	<0.0001	698 (55)
Mild cognitive impairment, *n* (%)	344 (51)	229 (38)	<0.0001	573 (45)
Moderate cognitive impairment, *n* (%)	7 (1)	2 (0.3)	0.129	9 (1)
**Previous CV history**
Myocardial infarction, *n* (%)	428 (63.6)	0 (0)		
Percutaneous coronary intervention, *n* (%)	472 (70)	0 (0)		
CABG, *n* (%)	246 (37)	0 (0)		
Stroke, *n* (%)	51 (8)	0 (0)		
Arrhythmia, *n* (%)	162 (24)	0 (0)		
Defibrillator, *n* (%)	37 (6)	0 (0)		
Pacemaker, *n* (%)	24 (4)	0 (0)		
**Laboratory data**
Systolic blood pressure (mmHg)	140 (127–154)	142 (129–155)	0.074	141 (127–154)
Diastolic blood pressure (mmHg)	69 (60–77)	73 (65–81)	<0.0001	71 (63–79)
Total cholesterol (mmol/l)	3.5 (3.0–4.0)	4.8 (4.1–5.6)	<0.0001	4.0 (3.3–5.0)
LDL cholesterol (mmol/l)	1.9 (1.6–2.4)	3.0 (2.4–3.7)	<0.0001	2.3 (1.8–3.2)
HDL cholesterol (mmol/l)	1.1 (1.0–1.4)	1.4 (1.2–1.7)	<0.0001	1.3 (01.0–1.6)
Triglycerides (mmol/l)	1.5 (1.1–2.0)	1.4 (1.0–2.0)	0.059	1.5 (1.1–2.0)
Glucose (mmol/l)	5.9 (5.5–6.8)	5.7 (5.3–6.2)	<0.0001	5.8 (5.4–6.4)
Insulin (pmol/l)	75 (47–119)	59 (37–91)	<0.0001	66 (41–104)
C-reactive protein (mg/l)	1.2 (0.6–2.8)	1.4 (0.6–2.9)	0.198	1.3 (0.6–2.9)
IL-6 (pg/ml)	2.1 (1.4–3.2)	1.6 (1.1–2.2)	<0.0001	1.8 (1.2–2.7)
sIL-6Rα (ng/ml)	24.6 (20.1–29.5)	24.6 (18.8–30.2)	0.722	24.6 (19.6–29.8)
**Medication**
Antiplatelets, *n* (%)	594 (88)	138 (23)	<0.0001	732 (57)
Aspirin monotherapy, *n* (%)	485 (72)	133 (22)	<0.0001	618 (48)
P2Y_12_ inhibitor monotherapy, *n* (%)	26 (4)	5 (1)	0.0004	31 (2)
Dual antiplatelet therapy, *n* (%)	83 (12)	0 (0)	0.003	83 (7)
Anticoagulants, *n* (%)	93 (14)	3 (1)	<0.0001	96 (8)
Antihypertensive, *n* (%)	595 (88)	250 (41)	<0.0001	845 (66)
Cholesterol-lowering medications, *n* (%)	615 (91)	264 (44)	<0.0001	879 (69)
Antidepressants, *n* (%)	78 (12)	69 (11)	0.901	147 (11)

*Values are means (SD) if normally distributed or median (25th−75th percentile) otherwise. 10-year cardiovascular (CV) risk was calculated using the Framingham Risk Score. Scored out of a possible total of 30 points, severe, moderate, mild, and no cognitive impairment, respectively reflect MoCA scores <10, between 10 and 17, between 18and 26, and ≥26. BMI, body mass index; CABG, coronary artery bypass grafting; CV, cardiovascular; HDL, high-density lipoprotein; IL-6, interleukin-6; LDL, low-density lipoprotein; sIL-6Rα, soluble interleukin 6 receptor alpha*.

**Figure 1:**
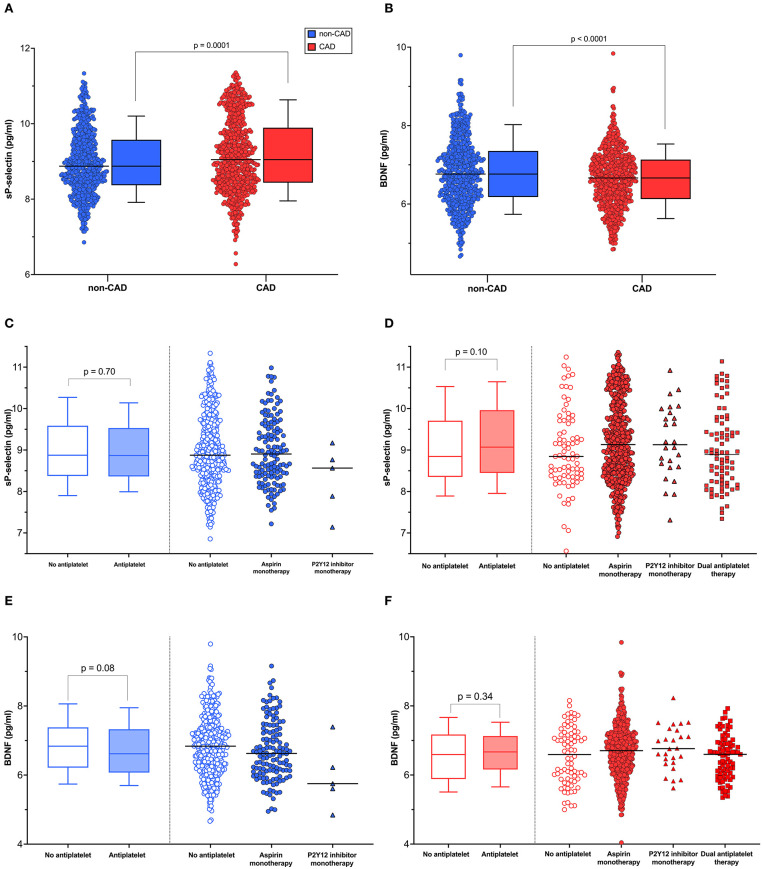
Distribution of sP-selectin levels and BDNF concentrations. **(A)** sP-selectin and **(B)** BDNF levels in CAD group (*n* = 673) and non-CAD group (*n* = 607). **(C,D)** sP-selectin and **(E,F)** BDNF levels distributed among users and non-users of antiplatelet therapy. The lines represent the median value; the box marks the 75th and the 25th percentiles; and whiskers indicate the 90th and 10th percentiles. An independent *t*-test was used to test for significant log-transformed sP-selectin and BDNF levels difference.

As shown with the log-transformed data in Figure 1A, individuals with CAD presented with higher platelet activation levels as measured by sP-selectin (8,504 (4,611; 19,815) vs. 7,150 (4,312; 14,388) pg/ml, *p* = 0.0001), despite more commonly receiving antiplatelet therapy (88% of CAD participants and 23% of non-CAD participants received antiplatelets, *p* < 0.0001). Plasma BDNF levels [784 (458; 1,253) vs. 866 (483; 1.563) pg/ml, *p* < 0.0001] (Figure 1B), and MoCA scores [26 (20, 21) vs. 27 (22, 23), *p* < 0.0001] were lower in CAD vs. non-CAD participants.

### Higher Platelet Activity Correlates With Higher BDNF Levels: Moderation by CAD Status

**Figure 2:**
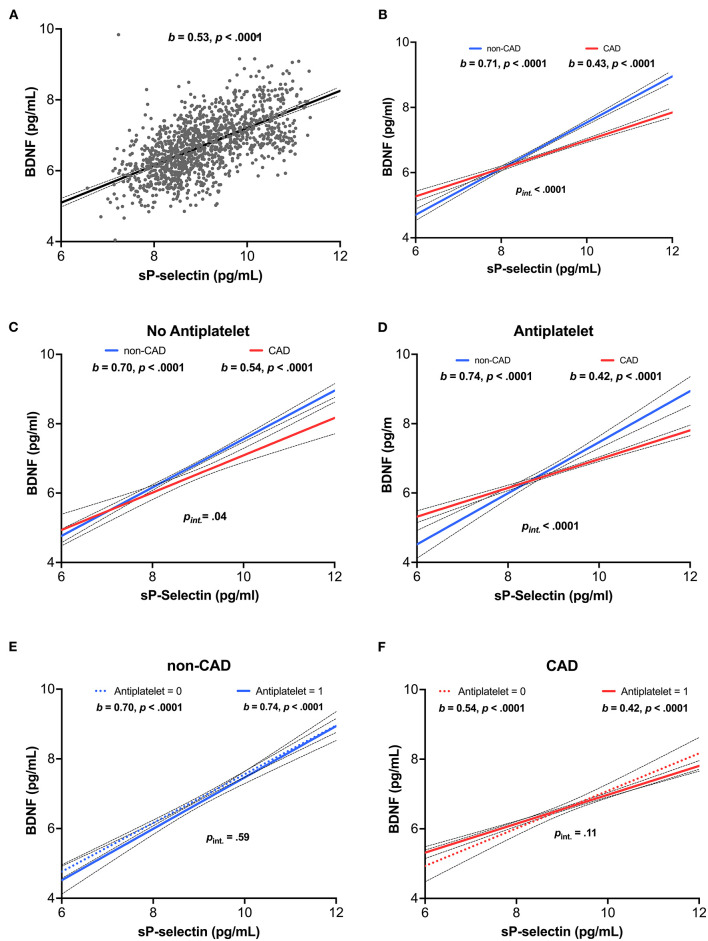
Relationship between platelet activity and BDNF concentrations. **(A)** Association of sP-selectin to BDNF concentrations in the overall population (*n* = 1,280). **(B)** Interaction with CAD status. Association of sP-selectin to BDNF concentrations in subjects **(C)** without and **(D)** with antiplatelet therapy. **(E,F)** Interaction with antiplatelet medication. sP-selectin and BDNF concentrations were log-transformed to account for a skewed distribution. b: unstandardized coefficient.

Regression analysis adjusted for age and sex in the whole population showed sP-selectin to be significantly correlated with BDNF levels (b = 0.53, *p* < 0.001, Figure 2A; Supplementary Table 1). A significant CAD status by sP-selectin interaction predicted BDNF plasma levels (b = −0.28, *p* < 0.0001), even after adjustment for covariates (Supplementary Table 1). More specifically, correlations between sP-selectin and BDNF were smaller in CAD participants (b = 0.43, *p* < 0.001) as compared with non-CAD participants (b = 0.71, *p* < 0.001, Figure 2B).

We considered that the moderating effect of the group could be due to antiplatelet therapy, which was frequent in CAD participants vs. non-CAD participants. Regardless of whether or not an antiplatelet drug was taken, the moderating effect of CAD remained significant. The correlation between sP-selectin and plasma BDNF was lower in CAD participants (without antiplatelets: b = 0.54, *p* < 0.0001, Figure 2C; with antiplatelets: b = 0.42, *p* < 0.0001, Figure 2D) than in subjects without CAD (without antiplatelets: b = 0.70, *p* < 0.0001, Figure 2E; with antiplatelets: b = 0.74, *p* < 0.0001, Figure 2F). The group interaction effect remained significant in the analysis with (*p*_interaction_ <0.0001, Figure 2D) and without antiplatelet therapy (*p*_interaction_ = 0.04, Figure 2C). In addition, no significant interaction of antiplatelet drugs and sP-selectin was found either for CAD participants (*p*_interaction_ = 0.11, Figure 2F) or non-CAD participants (*p*_interaction_ = 0.59, Figure 2E). Finally, there was no significant difference in either plasma sP-selectin (Figures 1C,D; Supplementary Table 2) or BDNF levels (Figures 1E,F; Supplementary Table 2) between individuals taking antiplatelet therapy or not.

### Higher BDNF Levels Are a Reverse Mediator in the Negative Relationship Between Platelet Activity and Cognitive Function

**Figure 3:**
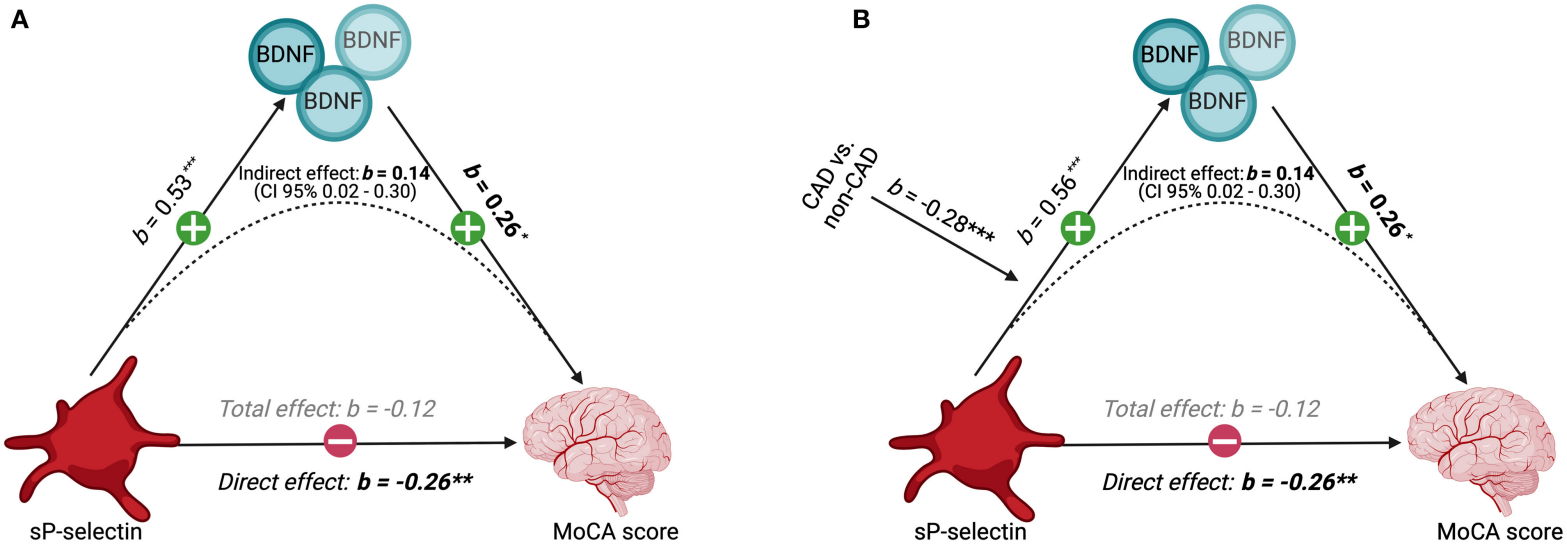
Interplay between platelet activity, BDNF levels, CAD status, and cognitive performance. **(A)** Mediation model between sP-selectin (independent variable), plasma BDNF concentrations (mediator), and the MoCA score (dependent variable) in the overall population (*n* = 1,280), **(B)** moderated by CAD status, showing unstandardized coefficients. sP-selectin and BDNF concentrations were log-transformed to account for a skewed distribution.

In the overall sample, the association of sP-selectin levels and MoCA score was not significant (total effect: b = −0.12, *p* = 0.13). However, once BDNF was included within the regression as a mediator, higher platelet activity became negatively associated with cognitive function (direct effect: b = −0.26, *p* = 0.01; Figure 3A). Indeed, the model showed that higher platelet activity was associated with higher plasma BDNF concentrations (b = 0.53, *p* < 0.0001), and higher BDNF concentrations were associated with better cognitive function (b = 0.26, *p* = 0.03). Thus, in a case of inconsistent mediation, the indirect effect of sP-selectin levels on the MoCA score through BDNF (b = 0.14, CI = 0.02–0.30) was in opposition to the direct effect of sP-selectin on the MoCA score (b = −0.26, *p* = 0.01), resulting in the overall non-significant association between platelet activity and cognitive function.

### CAD Moderates the Mediation Relationship Between Platelet Activity, Plasma BDNF, and Cognitive Function

CAD status significantly influenced the effect of sP-selectin on plasma BDNF levels (b = −0.28, *p* < 0.0001). Specifically, the relationship between sP-selectin and BDNF concentrations was stronger for individuals without CAD (b = 0.71, *p* < 0.0001) than for CAD participants (b = 0.43, *p* < 0.0001) (Figure 3B). This resulted in an indirect effect of sP-selectin levels on the MoCA score through BDNF in CAD participants (b = 0.11, CI = 0.01–0.22) that was significantly weaker than in non-CAD participants (b = 0.18, CI = 0.02–0.35), with a difference of −0.07 (−0.14 to −0.01) (Figure 3B).

## Discussion

In the present study, we have shown: (1) patients with CAD exhibited lower plasma BDNF levels and higher soluble P-selectin levels than their non-CAD counterparts, despite the use of antiplatelet therapy; (2) soluble P-selectin levels were correlated with plasma BDNF levels, an association that was significantly influenced by the presence of CAD, and (3) plasma BDNF was a reverse mediator in the negative relationship between platelet activation and cognitive function.

### Relationship Between BDNF and CAD

Patients with CAD exhibited significantly lower plasma BDNF levels than their non-CAD counterparts in the current study. This is in line with previous investigations that have also reported lower plasma BDNF levels in patients suffering from CAD (24, 25). Importantly, low serum BDNF levels in patients with CAD were shown to associate with an increased risk of adverse cardiovascular events and mortality (26). As platelets are the largest peripheral reservoir of BDNF, it would be expected that circulating BDNF levels would be a reflection of platelet activity, and it is indeed what was seen here and elsewhere (20, 22, 27). What was perhaps surprising was that the use of antiplatelet therapy, which reduces the risk of thrombotic complications through inhibition of platelet activity, did not significantly modulate circulating BDNF levels. This could be explained by the fact that most patients in our study were receiving aspirin monotherapy, and very few were treated with dual antiplatelet therapy with a P2Y_12_ inhibitor. Indeed, Stoll et al. have shown that administration of clopidogrel, but not aspirin, significantly reduced circulating BDNF levels in healthy volunteers (28).

It was noteworthy that CAD *per se* was associated with lower BDNF levels in our study, independent of antiplatelet treatment, and that the presence of CAD significantly influenced the relationship between platelet activity and circulating BDNF levels. Indeed, while we have found that P-selectin levels were positively correlated with BDNF levels in both CAD and non-CAD participants, the association was more pronounced in participants without CAD. Given the cross-sectional nature of our study, we cannot infer whether lower BDNF levels confer greater cardiovascular risk, or whether the presence of CAD lowers BDNF levels through an unknown mechanism. Interestingly, analyses from the Framingham Heart Study found lower BDNF levels to precede incident stroke, cardiovascular disease and mortality (21, 29). Longitudinal follow-up of our cohort is planned to address the directionality of effect between lower BDNF levels and CAD.

### Relationship Between Platelet Activity, BDNF, and Cognitive Performance

In animal models of stroke, local application of BDNF has been shown to improve recovery of neuronal function after ischemic injury (23). In humans, several lines of evidence support the positive association between higher circulating levels of BDNF and better cognitive health. Indeed, lower BDNF levels have been associated with lower cognitive performance (30), and patients with lower BDNF levels have been found to be at greater risk of progressing toward dementia (13). The ability of platelets to regulate BDNF bioavailability in circulation renders them potentially important vectors of BDNF to the site of cerebrovascular injury (9, 10).

In contrast, higher platelet activity has been reported in patients with cognitive impairment and dementia (2, 31). Increased platelet activity could contribute to cognitive dysfunction through several mechanisms. For example, platelet activation is associated with progression of carotid artery disease (32), while aggregating platelets contribute to vasoconstriction with hypoperfusion (2, 33, 34). Conversely, platelets help maintain vascular integrity and heal wounds (35). They are versatile cells that contain an abundance of growth factors and vascular mediators (36), which can be released upon platelet activation at sites of vascular injury (37, 38). Like these bioactive mediators, the platelet release of BDNF could therefore be beneficial in the healing of vascular and nerve damage in cerebrovascular disease.

Indeed, we found evidence that plasma BDNF partially mediated the relationship between platelet activity and the MoCA score, and this mediating effect was moderated by the presence of CAD. Surprisingly, while platelet activity was not significantly related to MoCA scores in analyses that did not include BDNF, the latter's inclusion as a mediator revealed a negative association between platelet activity and cognitive health. Moreover, analyses suggested that plasma BDNF mitigated the adverse effects of platelet activity on MoCA scores in a case of inconsistent mediation. It therefore appears possible that platelet activation may have two opposing effects on cognitive health. More specifically, our model suggests that platelet activity could have a positive effect via the release of BDNF at the site of cerebrovascular injury, while a deleterious effect could occur via an increased risk of thrombosis, resulting in a scenario of inconsistent mediation. Interestingly, the mediating effect of plasma BDNF on platelet activity related cognitive decline was significantly lower in CAD patients than in subjects without CAD. Knowing that patients with CAD are at higher risk of cognitive impairment (39), the mechanisms behind this weakened protective effect of BDNF on platelet-related cognitive deterioration is worthy of further investigation. Future studies should examine *in vitro* the content and release of BDNF from platelets isolated from CAD and non-CAD individuals, to have a better understanding of mechanisms regulating platelet BDNF secretion in health and disease.

### Limitations

Our results should be interpreted with caveats in mind. First, because of the cross-sectional design of the study, it is not possible to infer a direction or a causal link between BDNF levels, platelet markers and cognitive decline. Secondly, although platelets are the largest extra-cerebral reservoir of BDNF, other sources of BDNF such as endothelial cells, immune cells or the skeletal muscle, could be responsible for the different levels seen in this study (40–43). The relative contribution of extra-platelet sources to plasma BDNF levels is unknown. For example, while BDNF protein is highly expressed in human skeletal muscle and is increased after exercise, muscle-derived BDNF appears not to be released into circulation (42). Indeed, almost all BDNF contained in blood appears to be stored in platelets, and to be secreted upon platelet activation (10, 11). This is consistent with the high level of correlation between P-selectin and BDNF levels seen herein, but does not preclude other contributors to circulating BDNF levels. Similarly, although platelets are the major source of plasma sP-selectin, activation of endothelial cells could also have contributed to circulating levels (44–46). We cannot exclude false negatives (type II errors), due to the limited statistical power in subgroup analyses involving use of antiplatelets, requiring replication in larger samples. We have used plasma concentrations as a reflection of *in vivo* platelet activation and BDNF levels. Caution is required when comparing studies having measured BDNF levels in plasma vs. serum, as serum production induces *in vitro* platelet activation and massive release of BDNF by a factor >100 (9, 10). As such, studies using serum could be seen as a reflection of platelet reserves of BDNF, while studies using plasma could be seen as an *in vivo* reflection of circulating BDNF levels. Whether circulating vs. platelet-borne BDNF levels have the same biological function remains to be elucidated. Finally, our sample was constituted mainly of Caucasian francophones and it is not certain to what extent results apply to individuals of different ethnicity.

## Conclusion

In summary, our results suggest that the relationship between platelet activity and cognitive health is complex. In the context of vascular disease, where potential vascular damage to the cerebral microvasculature may lead to maladaptive platelet activation and thrombosis, the nefarious effects of cerebral thrombi on cognitive health are anticipated. However, future models should integrate the effects of biologically-active molecules secreted by platelets at the site of vascular injury, as platelet activation may also bring needed neuronal and angiogenic growth factors essential for recovery and may thus actively contribute to vascular healing. Approaches to balance the tipping point between hemostatic and maladaptive platelet activation are needed.

## Data Availability Statement

The raw data supporting the conclusions of this article will be made available by the authors, without undue reservation.

## Ethics Statement

The studies involving human participants were reviewed and approved by Research Ethics Committee of the Montreal Heart Institute [2011-202 (11-1313)]. The patients/participants provided their written informed consent to participate in this study.

## Author Contributions

JCB has performed assays and collected data, analyzed and interpreted data, and wrote the manuscript. VB, JLB, LS, and MW have performed assays and collected data, analyzed and interpreted data, and critically revised the manuscript. MCB has performed statistical analyses. DB has supervised biobank sample access. HC has critically revised the manuscript. BDA and ML have overseen the research group, designed the research, obtained funding, analyzed and interpreted data, and critically revised the manuscript. All authors have read and approved the final manuscript.

## Funding

This work was supported by the Canadian Institutes of Health Research (MOP #111015 awarded to BDA), the Canada Foundation for Innovation Leaders Opportunity Fund (32797 awarded to ML), and the Montreal Heart Institute Foundation (2018-1581 awarded to ML/2018-1681 awarded to BDA). JCB and LS were supported by Doctoral Training Awards from the Fonds de recherche du Québec en Santé (FRQS). JLB was supported by summer research scholarship from the Faculté de pharmacie de l'Université de Montréal. HC was supported by a Foundation Grant from the Canadian Institutes for Health Research, the Weston Foundation, and the Baycrest Health Sciences Foundation. ML was supported by the FRQS Junior 1 Research Scholarship (33048); and is a Canada Research Chair in Platelets as biomarkers and vectors (950-232706).

## Conflict of Interest

HC has participated as a site PI in pharmaceutical trial activities in the past 5 years sponsored by: Hoffmann-La Roche Limited, TauRx, Lilly, Anavex Life Sciences, Alector LLC, and Immunocal site investigator for trials; and is Scientific Director for the Canadian Consortium on Neurodegeneration in Aging, which receives partner support from a set of partners including industry: Pfizer Inc., Lilly, and Sanofi. ML has received speaker fees from Bayer; has participated in industry-funded trials from Idorsia; has served on advisory boards for Servier and Orimed Pharma; and has received in-kind and financial support for investigator-initiated grants from Leo Pharma, Roche Diagnostics, Aggredyne, and Fujimori Kogyo. The remaining authors declare that the research was conducted in the absence of any commercial or financial relationships that could be construed as a potential conflict of interest.

## Publisher's Note

All claims expressed in this article are solely those of the authors and do not necessarily represent those of their affiliated organizations, or those of the publisher, the editors and the reviewers. Any product that may be evaluated in this article, or claim that may be made by its manufacturer, is not guaranteed or endorsed by the publisher.

## Abbreviations

CAD: Coronary Artery Disease
BDNF: Brain-Derived Neurotrophic Factors
P-selectin: soluble P-selectin
MoCA: Montreal Cognitive Assessment
BMI: Body mass index.
