# Supplementary material for: Brain-Derived Neurotrophic Factor Mitigates the Association Between Platelet Dysfunction and Cognitive Impairment
Source: Front Cardiovasc Med. 2021 Sep 7;8:739045. doi: 10.3389/fcvm.2021.739045 (PMC8452906; doi:10.3389/fcvm.2021.739045)
Supplement: Supplementary file 1 [file Data_Sheet_1.PDF]

Supplementary Material to:

Brain-Derived Neurotrophic Factor mitigates the association between platelet dysfunction and cognitive impairment

Jean-Christophe Bélanger[1,2], Véronique Bouchard[1,3], Jessica Le Blanc[1,2], Louisia Starnino[1,3], Mélanie Welman[1], Malorie Chabot-Blanchet[5], David Busseuil[1], Howard Chertkow[6,7], Bianca D'Antono[1,8*], Marie Lordkipanidzé[1,2*]

[*] These authors have contributed equally and share last authorship.

[1] Research Center, Montreal Heart Institute, Montreal, Qc, Canada

[2] Faculty of Pharmacy, Université de Montréal, Montréal, Qc, Canada

[3] Psychology Departement, Faculty of Human Sciences, Université du Québec à Montréal, Montreal, Qc, Canada

[5] Montreal Health Innovations Coordinating Center, Montreal, Qc, Canada

[6] Baycrest Health Sciences and the Rotman Research Institute, University of Toronto

[7] Departement of Medicine (Neurology), University of Toronto, On, Canada

[8] Psychology Departement, Faculty of Arts and Sciences, Université de Montréal, Montreal, Qc, Canada

1 Supplementary Tables

S-Table 1. Hierarchical linear regression model of plasma BDNF levels

**Variable**	**R^2**	**ΔR^2**	**b**	**F**	**p**
**Step 1**	0.005			3.1	0.046
Sex			-0.100		0.032
Age			-0.004		0.201
**Step 2**	0.375	0.371		255.6	0.0001
Sex			-0.140		0.0001
Age			-0.005		0.049
sP-selectin			0.529		0.0001
**Step 3**	0.398			210.7	0.0001
Sex			-0.045		0.245
Age			-0.002		0.358
sP-selectin			0.542		< 0.0001
CAD			-0.265		< 0.0001
**Step 4**	0.423	0.025		186.5	0.0001
Sex			-0.046		0.219
Age			-0.003		0.270
sP-selectin			0.708		0.0001
CAD			2.265		0.0001
sP-selectin X CAD			-0.279		0.0001

S-Table 2. The effect of antiplatelet therapy on platelet activity and BDNF levels

Group	Therapy	n	sP-selectin		log sP-selectin				BDNF		log BDNF			
			Median	IQR	Mean	SD	t-test	p	Median	IQR	Mean	SD	t-test	p
non-CAD	No antiplatelet	469	7150	4340-14548	8.98	0.87	0.38	0.36	931	501-1603	6.84	0.86	1.79	0.07
	Antiplatelet	138	7105	4290-13769	8.94	0.82			740	437-1498	6.69	0.87		
CAD	No antiplatelet	79	6945	4243-16435	9.00	0.96	1.67	0.75	766	360-1289	6.55	0.81	0.97	0.34
	Antiplatelet	594	8694	4667-21181	9.19	0.99			787	473-1245	6.40	0.75		

IQR: Interquartile Range (25th - 75th percentile), SD: Standard Deviation
